# A descriptive social and health profile of a community sample of adults and adolescents with Asperger syndrome

**DOI:** 10.1186/1756-0500-3-300

**Published:** 2010-11-12

**Authors:** Myles Balfe, Digby Tantam

**Affiliations:** 1 Centre for the Study of Conflict and Reconciliation, School of Health and Related Research, University of Sheffield, Regent Court, 30 Regent Street, Sheffield, S1 4DA, UK

## Abstract

**Background:**

Little is known about the health and social profile of adolescents and adults with Asperger syndrome (AS) living in the community. We conducted a study to describe the living, employment and psycho-social situation of a community sample of forty two adults and adolescents with AS, and to describe these indivdiuals' experiences of accessing health services and taking medication.

**Findings:**

Most respondents (including those over eighteen years of age) lived at home with their parents. Most had trouble reading and responding to other people's feelings, and coping with unexpected changes. Difficulties with life skills, such as cleaning, washing and hygiene were prevalent. The majority of respondents were socially isolated and a large minority had been sexually or financially exploited. Almost all respondents had been bullied. Mental health problems such as anxiety or depression were common. 30% of respondents said that they regularly became violent and hit other people and 15% had attempted suicide. More positively, the majority of respondents felt that they could access health services if they had a health problem.

**Conclusions:**

The results of this study suggest a relatively poor social and health profile for many people with Asperger syndrome living in the community, with high levels of social problems and social exclusion, and difficulties managing day to day tasks such as washing and cleaning; these findings support the results of other studies that have examined psycho-social functioning in this group.

## Background

Asperger syndrome (AS), or High Functioning Autism (HFA), is a neurobiologic disorder with a strong heritability [1,2]. It is defined by qualitative impairments in social communication, interaction, and by the presence of repetitive interests. Although delay in language or cognitive development is given as a differentiating feature of Asperger syndrome from Autistic disorder, the adult status of some people with a childhood developmental history meriting the diagnosis of Autistic disorder (the high-functioning autism group) may become indistinguishable from adults who have met the criteria of Asperger syndrome in childhood. Clinically, the main differentiation between the high- and low-functioning groups in adulthood is whether or not the person with an autistic spectrum disorder has an awareness of their difficulties, and their difference from others [3]. This criterion is difficult to operationalize in a research study, and the best proxy measure is probably the absence of significant learning difficulty, with the threshold conventionally set at an IQ of 70 or greater.

Research with clinical populations of people with Asperger syndrome suggests that this group often experiences significant health and social difficulties [4]. Specific domains of functioning where people with Asperger syndrome can face difficulties include, but are not limited to, social skills (making and maintaining friendships, carrying on conversations, talking about a range of matters) [5], empathizing with others, anger management, dealing with bullying and teasing, handling developmental transitions [6], and with mental health problems such as anxiety and depression [7,8]. Help with employment is also often required- some studies have found that only 12% of adults with AS are in paid employment [9].

Though our understanding of the health and social profiles of people with Asperger syndrome has increased, there are still gaps in our understanding. We know little about the health and social situations of adults with the condition [6]. And we know little about people with Asperger syndrome who do not routinely encounter clinical services or who are not members of autistic societies (both of which are channels through which people with Asperger syndrome are routinely recruited for research projects). When people with AS from the community are recruited for research studies their Asperger syndrome is often not confirmed [10], making it unclear whether or not participants in these studies do or do not have Asperger syndrome. The disadvantage of focusing on clinical populations is that this may lead to selection bias. The health and social profiles of people with AS who attend clinical services may be different in severity and type from those who do not routinely attend clinical services.

This paper reports the findings of a community-based study designed to describe the health and social profiles of a group of adolescents and adults with Asperger syndrome in the UK. In contrast to many previous studies, we did not rely solely on charitable [11] or professional ascertainment (although we did contact a wide range of professionals), but invited adolescents (aged 13 or over) and adult men and and women to come forward through a public and professional awareness campaign if they had a diagnosis of Asperger syndrome. We attempted to screen all respondents using the Autism Diagnostic Interview (ADI-R) [12] and assess their IQ using a standard IQ screening measure (the Wechsler Abbreviated Scale of Intelligence) [13].

All respondents completed a questionnaire asking them to describe their living, employment and social situations, and their experiences of accessing health services and taking medicaion. Results were analyzed using simple descriptive statistics.

## Methods

### Recruitment

We employed a number of strategies to recruit potential respondents. We sent letters to every headmaster, general practice, neurologist, paediatrician and community paediatricians and psychiatrist in the city in which the study was conducted, inviting them to bring the study to the attention of any of their patients, pupils or clients who had Asperger syndrome or high functioning autism and who were thirteen years or age and above. Similar recruitment methods have been used in previous studies. These methods, however, are not necessarily suitable for the recruitment of adults and school leavers as adults and older adolescents are not 'captive populations' in the same way that children are. We therefore designed posters to invite adults and older adolescents to contact us if they had Asperger syndrome or HFA. We placed these posters in supermarkets, shops, cinemas, post offices, libraries, pubs, workplaces, student noticeboards, employment assistance agencies and doctor's surgeries throughout the city. We wrote articles for two local newspapers, and gave an interview on the radio about the study. We recruited respondents through the local parents support group. We placed information about the project in the local employment agency for people with autism and sent information about the study to social workers, care workers and disability workers throughout the city.

Sixty eight respondents contacted us saying that they had Asperger syndrome or HFA and wished to take part in the study. We asked all of these respondents to complete questionnaires describing their health and social situations (more on the questionnaire is said below). Sixty eight questionnaires were returned. Twenty three of these questionnaires were excluded when it emerged that the respondents who completed them did not have clinical diagnoses of AS/HFA (these twenty three respondents believed that they could have AS/HFA, but did not have diagnoses to support their beliefs). Forty five respondents' questionnaires were therefore deemed potentially eligible for inclusion. We asked each of these forty five respondents if they would be willing to let us complete ADI-R [12] interviews with their parents, and WASI [13] IQ assessments with themselves. Seventeen respondents agreed. The WASI has good predictive accuracy when used with indivdiuals with high-functioning autism [13]. The ADI-R is a developmental history interview that is conducted with the parents/caregivers of an individual being evaluated for Asperger syndrome. It focuses on language and communications, reciprocal social interactions and restrictive, repetitive and stereotyped behaviours and interests. The person who administered the ADI-R had an ADI-R inter-rater reliability score of above 90%. A qualified trainer at the Autism Research Centre in the University of Cambridge assessed the first ADI-R interview that the person completed; the trainer agreed with 90% of the interviewer's ADI-R codings. Subsequent ADI-R interviews were coded solely by the interviewer. We confirmed fourteen respondents' Asperger syndrome using the ADI-R and the WASI (two respondents were excluded because their ADI-R scores fell below the diagnostic threshold, and one because his IQ was below 70). Though we were unable to conduct ADIR/IQ assessments with twenty eight respondents (these respondents either did not reply to our letter, or said that they did not wish for us to talk to their parents), we did either a). obtain documentation from them supporting their stated diagnoses of Asperger syndrome or high-functioning autism (e.g. doctor's certificates, educational statements) or b). telephone their parents/caregivers/partners to confirm their diagnoses. Forty two questionnaires were therefore ultimately deemed eligible for inclusion in the study.

### The questionnaire

The questionnaire was a multi-item instrument that was designed to completed in approximately twenty minutes. It investigated respondents': demographic profiles; living situations; social difficulties; social geographies; experiences of health and health services; and experiences of ostracism and exploitation (a detailed list of items included in the questionnaire is found in additional file 1). Items included in the questionnaire were identified via a). a literature review and b). through interviews with seven people with with AS and their parents. These interviewees were asked to identify areas of functioning where they believed people with Asperger syndrome faced difficulties in their day-to-day lives, or which they believed were a cause of conern for people with AS and/or their caregivers. The questionnaire was a forced-choice instrument: respondents were asked to tick 'yes/no' to all questions.

We encouraged respondents to seek help from family members or care givers if they experienced difficulties completing the questionnaire. Analysis of the consent forms that accompanied all completed questionnaires revealed that most respondents had assistance in completing the survey.

Data were analysed using simple descriptive statistics in SPSS 12. The local research ethics committee granted the project ethical approval and all respondents gave written informed consent to take part in it.

## Results

The majority of respondents were male (88%) and white (92%). 8% of respondents were from a Black Caribbean background. The mean age of respondents was 26.21 years (standard deviation 11.9 years, range 13-64). Sixteen respondents were under eighteen years of age and twenty six were above eighteen years of age.

Results are presented in Tables 1, 2 and 3. Table 1 presents respondents' living and employment situation and social geographies, Table 2 respondents' health profiles and Table 3 respondents' interpersonal and social problems. Several items have '18+' written after them: this means that the particular percentage figure for that item was generated from the answers of respondents who were aged eighteen years of age and above.

**Table 1:** Living and employment situation and social geographies

Living situation (for 18+)	With parents:	69%
	In own home:	14%
	Other (e.g. married):	17%
**Places regularly visited:**	Library:	53%
	Cinema:	48%
	The pub:	36%
	Community centres:	10%
	Nightclub:	0%
	Never leave home:	7%

**Employment situation (for 18+):**	Paid work:	21%
	Paid work with support:	7%
	Voluntary work:	4%
	Voluntary work with support:	7%
	Day care:	17%
	No employment activity:	15%

**Table 2:** Health profile

Experiences of medication	Currently taking medication for health problem:	54%
	Experienced side-effects from medication?:	78%
	Not informed of what medication would do?:	10%
	Not informed of side-effects of medication?:	30%
**Physical and mental health problems:**	Poor eyesight:	24%
	Poor hearing:	24%
	Involuntary movements:	36%
	Genetic disorder:	16%
	Neurological problems:	16%
	Anxiety:	51%
	Body image problem:	42%
	Depression:	35%
	Memory problems:	48%
	Angered easily:	84%
	Often hit people:	31%
	Suicidal thoughts:	40%
	Attempted suicide in past:	15%
	Alcohol problems:	15%
	Street drug problems:	10%
	Trouble with the police:	15%

**Health services**	Able to get help from health services?:	92%
	Been in contact with health services in past year?:	82%
	Been in contact with social services in past year?:	51%
	Receiving state benefits?:	57%
	Carer receives benefits?:	31%
	Able to get help if have money problems?	30%

**Table 3:** Interpersonal and social problems

Have difficulties:	Reading other people's feelings:	91%
	Responding to people's feelings:	86%
	Trouble showing own feelings:	42%
	Trouble managing time and planning:	61%
	Trouble coping with unexpected changes:	87%
	Stopping spending time on interests:	84%
	Managing money:	67%
	Cooking:	60%
	Tidying and cleaning:	52%
	Washing and showering:	31%
	Doing well in jobs interviews:	40%
	Getting to places on time:	31%
	Reading books and newspapers:	71%
	Writing neatly and legibly:	65%
	Living independently without support (18+):	67%
	Using public transport:	64%
	Switching tasks:	85%
**Social problems**	Been bullied:	95%
	Feel misunderstood:	63%
	Feel left out of things:	77%
	Feel put down by others:	62%
	Feel sexually frustrated:	56%
	Been sexually or financially exploited:	40%
	Have distressing family problems:	31%

The results presented in Table 1, 2 and 3 are based on summary statistics of all 42 respondents. Because we were able to confirm the diagnosis of only fourteen respondents we compared the answers of these respondents with the answers of those respondents whose diagnosis we were unable to confirm through ADI-R interviewing. The results were similar and no statistically significant differences at the p < .005 level were were found between the two groups using chi-square analysis in SPSS; additional file 2 shows how similar both the ADI-R confirmed and unconfirmed groups are on key survey items that tap into AS diagnostic criteria, and help justify combining the two groups into one larger AS group.

## Discussion

This is one of the the few studies to describe the health and social care situations of a community sample of adolescents and adults with Asperger syndrome in the UK. The study is original in the approach it used to recruit respondents. We did not recruit participants through charitable or clinical channels, but rather through a general media awareness campaign. As little material has been published on adults at the higher functioning end of the autistic spectrum [7], the information collected here helps to address some of the gaps in our current knowledge of adults and adolescents with AS.

Several points are worth highlighting from the study's results. Social problems were prevalent in this sample. Almost all respondents had been bullied. A substantial number of respondents had either been sexually or financially exploited. The majority of respondents felt either left out of things or put down by other people. These findings echo those of previous studies that have examined the social challenges faced by adults on the autistic spectrum [14,15]. They describe a vulnerable, marginalized and socially excluded group [3], one characterized by social isolation and difficulties with social interactions [16].

Given this social alienation, and the social communication difficulties that respondents also described, it is perhaps unsurprising that respondents' social geographies (the places where they most regularly went on a daily basis) were mainly limited to spaces where social interaction with other people was kept at a minimum (such as libraries). No respondent, for example, ever described going to a nightclub. Almost one in ten respondents did not go out at all. A number of respondents said that they went to the pub on a regular basis. Conversations with parents and caregivers after ADI-R sessions, however, revealed that going to the pub was a process that was mainly driven by respondents' parents; while they were at the pub respondents mixed principally with their parents' friends. Previous studies too have found that individuals with AS/HFA often have limited social geographies [17,18]. Wainscot et al. [18], for example, found that secondary school pupils with AS often spend much more time than their peers do inhabiting places where they do not have interact with other people. In their prospective follow-up study of 70 males with Asperger syndrome, Cederlund et al. [19] found that 26% of their cases lived a 'very restricted life' and in another follow-up study Billstedt et al. [20] found that many people with AS lived 'fairly isolated lives'.

Most respondents over eighteen years of age continued to live in the parental home. This finding supports the results of a previous survey that was conducted by the United Kingdom National Autistic Society [11] which reported that 49% of adults with autism or AS still lived at home with their parents. Previous studies also support this study's finding that people with Asperger syndrome often require assistance with living independently [21]. Many remain unusually dependent upon their parents in a way that other individuals of the same age would not [22,10,23].

Only 21% of survey respondents over eighteen years of age were in some form of paid work. These figures are higher than those found in a recent study by the National Autistic Society in the UK [11] which found that only 12% of high functioning adults were in fulltime employment and higher than the employment rates in the first 46 patients that the second author systematically studied between 1980 and 1982, of whom 9% were employed [3], but it is still far lower than the average national employment rate. One question that we did not ask was what kinds of job were respondents working at; many people with AS have menial and often supervised jobs where they work for only a few hours a week or receive only the minimum wage [23]. Almost 20% of respondents described having no activity during the day. Difficulties in finding work may in turn have negative consequences for people with Asperger syndrome, for example for their mental health. As well as their difficulties with social skills, the difficulties that respondents experienced with reading and writing may also have negatively impacted on respondents' employment prospects.

Mental health problems were common. Half of respondents described regularly feeling anxious and a lesser, though still substantial number, described regularly feeling depressed, figures that are similar to those reported in previous surveys [24,11,25]; though comparing the anxiety and depression results of this study with those of these others should be done cautiously. Depression in the noted studies [24,11,25] was assessed using diagnostic instruments, whereas we used a much blunter and less sensitive instrument, i.e. the questions 'do you feel depressed', 'do you feel anxious'. Respondents, for example, may not have recognized symtoms of their own depression, which would have lead to depression being under-reported in this study. Tantam and Girgis [26] note that anxiety and anxiety-related disorders of clinically significant severity may be present in up to 40% of clinical samples. Anxiety and depression are often triggered in people with AS as a consequence of their struggles to understand complex social mileus and because of their awareness of their differences from others [27,28]. Anxiety may also be increased by experiences of bullying (as noted, most of the sample in this study were bullied) and by experiences of victimization (again, a substantial number of repsondents had been either financially or sexually exploited) [26]. Two fifths of respondents had thought about committing suicide at some point in the past, and 15% of respondents reported that they had attempted to kill themselves. Risk factors for suicide include childhood adversity, individual and personal vulnerabilities and exposure to stressful life events and circumstances [29]. People with Asperger syndrome match this risk profile [6].

Problems with aggression and violence were also common. Berney [8] notes that children with Asperger syndrome who have anxiety or mood problems are often more aggressive than those without, which may account for the high levels of violent behaviour reported in this study. Figures here match those supplied by Tantam [3] though exceed by some threshold figures supplied in other studies [30]. Other risk factors for violence in people with Asperger syndrome include impairments in interpreting nonverbal expressions, which can reduce empathy for other [6], and feeling isolated and powerless, which are again characteristics shared by this sample. Violent and aggressive behaviour by people with AS may be especially unsettling as it may be directed against vulnerable people rather than the people who triggered the violence, and the violence may be temporally removed from its trigger [26].

Respondents' experiences of health and social services were generally positive. Most respondents felt that they would be able to access health services if they had a problem. Two areas are of concern. One is that only 32% of respondents felt that would be able to get money if they ran into financial difficulties. Because of their interpersonal impairments, many respondents may have difficulties accessing and negotiating financial services. The difficulties that respondents had with writing and reading may intensify these difficulties. The other area of concern is the percentage of respondents who felt that they were not informed about the side-effects of their medication. Clinical experience informs us that patients often forget information that is shared with them verbally, and this is a group for whom this may be especially true (a number of respondents reported having memory problems). It may be useful to supply people with Asperger syndrome salient advice about their medication in both written and verbal forms. Overall, the figures for respondents in this study who reported taking medication were similar to other recent studies (54% in this study compared to 58% in Eaves and Ho's [23]). Adolescents and adults with ASD, including AS/HFA are a highly medicated population [31]. In Eaves and Ho's study [10] their respondents with AS were taking medication for seizures, allergies, behaviour modification, thyroid problems and sleep disturbances; a number of respondents in Eaves and Ho's study were on three or more drugs.

This study has a number of weaknesses. The small number of people who took part in the study prevented us from completing more complicated multivariate analysis and detecting statistically significant differences between subgroups of respondents. We found it difficult to confirm the diagnosis of many of respondents using the ADI-R (though we compensated for this by receiving diagnosis supporting information such as educational statements). Against this, most respondents who completed the health and social needs questionnaire had assistence from either a parent or a partner, meaning that the answers reported in each questionnaire are in effect the result of a consensus between reached two people. This hopefully serves to increase the objectivity of the information reported. Results present 'yes/no' answers for each item; as such results do not reveal the intensity that each survey item had for respondents. While many respondents said that they felt anxious, for example, some may have felt anxious all the time, whereas for others anxiety may have been more transient. We also did not capture socio-economic data on respondents. Finally, care should be taken when generalizing the results of this study. The numbers in the study are small, and attrition was significant. We could not confirm the diagnosis of a number of respondents using the ADI-R. We would urge other researchers to interpret this study's findings as a preliminary pilot guide encouraging further investigation of the items explored here, rather than definitive results for people with AS living in the community.

## Conclusion

The results from this study cannot be unreservedly generalized to everyone with Asperger syndrome. They do suggest, however, a relatively difficult social profile for at least some people with Asperger syndrome in the community, with high levels of social problems and social exclusion, and difficulties managing day to day tasks such as washing and cleaning; these findings support the results of other studies that have examined psycho-social functioning in this group [32,33]. Further, and larger, community studies are needed to replicate the findings of this study.

## Competing interests

The authors declare that they have no competing interests.

## Authors' contributions

MB analyzed the surveys and wrote the first draft of the manuscript. DB conceived of the study, participated in its design and coordination and revised the manuscript. All authors read and approved the final manuscript.

## Supplementary Material

Additional file 1 **Survey items**. Table containing list of questions from the survey Click here for file

Additional file 2 **Comparison of answers of respondents with and without confirmed ADI-R diagnoses**. Table comparing the answers of respondents with and without confirmed ADI-R diagnoses on key diagnostic items. Click here for file
