# Supplementary material for: A descriptive social and health profile of a community sample of adults and adolescents with Asperger syndrome
Source: BMC Res Notes. 2010 Nov 12;3:300. doi: 10.1186/1756-0500-3-300 (PMC2992545; doi:10.1186/1756-0500-3-300)
Supplement: Additional file 1 — Survey items. Table containing list of questions from the survey [file 1756-0500-3-300-S1.DOC]

**Appendix 1**

| - **Demographic profile** |
| --- |
| - - Gender (tick: male/female) |
| - - Ethnic group (tick: white/black/other) |
| - - Age (enter age) |
|  |
| **Where do you live? (for over eighteens only- tick yes/no)** |
| - - Group home with live in staff |
| - - With parents |
| - - In own home with no one else living there |
| - - Other (e.g. married) |
|  |
| **Do you find it difficult to: (tick ‘tick yes/no’ to all that apply)** |
| - - Read other people’s feelings |
| - - Respond to other people’s feelings |
| - - Show your own feelings |
| - - Plan and manage your time |
| - - Cope with unexpected change |
| - - Stop spending time on something you’re really interested in |
| - - Concentrate on one task |
| - - Respond to other people’s feelings |
| - - Show that you need help from other people |
| - - Switch tasks |
| - - Get on with other people of the same age as yourself |
| - - Manage money |
| - - Cook |
| - - Look after the house |
| - - Be on time |
| - - Clean and wash yourself |
| - - Use public transport |
| - - Do well in job interviews (for ever eighteens only) |
| - - Live independently without support (for over eighteens only) |
| - - Read book and newspapers. |
| - - Write legibly (have good handwriting) |
| - - Use public transport |
| - - Stop thinking about the past |
|  |
| **For over eighteens only, are you (tick all that apply):** |
| - - In paid work |
| - - In paid work with support |
| - - In education without support |
| - - In education with support |
| - - Doing voluntary work |
| - - Doing voluntary work with support |
| - - In day care |
| - - Involved in no activity during the day |
|  |
| - **Do you regularly go to (tick all that apply):** |
| - - The library |
| - - The cinema |
| - - The pub |
| - - Museums |
| - - Community centres |
| - - Nightclubs |
| - - Don’t really go out |
|  |
| **Tick ‘yes/no’ to all that apply:** |
| - - Are you able to get help if you have a health problem? |
| - - Have you been in contact with health services in the past year? |
| - - Have you been in contact with social services in the past year? |
| - - Are you receiving any state benefits? |
| - - If you have a carer, is he/she receiving any state benefits? |
| - - Are you able to get help if you are short on money? |
| - - Have you ever taken medication for a health problem? |
| - - Have you ever experienced side effects from taking this medication? |
| - - Have you ever been given medication but were not told what this medication would do to you? |
| - - Have you ever been given medication but were not informed about side effects? |
| - - Do you have poor eyesight? |
| - - Do you have poor hearing? |
| - - Do you have a problem with involuntary movements? |
| - - Do you have neurological problems? |
| - - Do you have a genetic disorder? |
| - - Do you often feel anxious? |
| - - Do you find yourself regularly becoming violent and hitting people? |
| - - Do you become angry very easily? |
| - - Have you ever thought about killing yourself? |
| - - Have you ever tried to kill yourself? |
| - - Have you ever had problems with alcohol? |
| - - Have you ever had problems with street drugs (e.g. heroin, cocaine) |
| - - Have you ever been in trouble with the police? |
| - - Do you have a problem with your body image? |
| - - Do you currently feel depressed? |
| - - Does your memory ever cause you problems? |
| - - Do you have any hobbies or activities? |
|  |
| **Have you ever (tick all that apply):** |
| - - Been bullied |
| - - Felt that other people don’t understand you |
| - - Felt left out of things |
| - - Felt that other people put you down |
| - - Wanted to have a sexual relatinship but were unable to have one |
| - - Felt sexually frustrated |
| - - Been financially exploited |
| - - Been sexually exploited |
| - - Had family problems that caused you distress |
|  |
