# Supplementary material for: A descriptive social and health profile of a community sample of adults and adolescents with Asperger syndrome
Source: BMC Res Notes. 2010 Nov 12;3:300. doi: 10.1186/1756-0500-3-300 (PMC2992545; doi:10.1186/1756-0500-3-300)
Supplement: Additional file 2 — Comparison of answers of respondents with and without confirmed ADI-R diagnoses. Table comparing the answers of respondents with and without confirmed ADI-R diagnoses on key diagnostic items. [file 1756-0500-3-300-S2.DOC]

| *Is this item a problem?* | *ADI-R diagnosis* | *Without ADI-R diagnosis* |
| --- | --- | --- |
| Reading other people’s feelings | 87% | 94% |
| Responding to other people’s feelings | 95% | 82% |
| Showing own feelings | 56% | 41% |
| Planning and time management | 70% | 75% |
| Coping with unexpected change | 88% | 88% |
| Spending too much time on interest | 88% | 88% |
| Concentrating on one task | 83% | 83% |
| Switching tasks | 93% | 83% |
| Getting on with people in different situations | 77% | 75% |
| Not understanding what other people say | 72% | 61% |
